# RePP Africa – a georeferenced and curated database on existing and proposed wind, solar, and hydropower plants

**DOI:** 10.1038/s41597-022-01922-1

**Published:** 2023-01-06

**Authors:** Rebecca Peters, Jürgen Berlekamp, Klement Tockner, Christiane Zarfl

**Affiliations:** 1 Department of Geosciences, Eberhard Karls University of Tübingen, 72076 Tübingen, Germany; 2 Institute of Environmental Systems Research, University of Osnabrück, 49076 Osnabrück, Germany; 3 Senckenberg Society for Nature Research, 60325 Frankfurt a. M., Germany; 4 Faculty of Biological Sciences, Goethe-University, 60323 Frankfurt a. M., Germany

**Keywords:** Databases, Energy supply and demand, Environmental impact

## Abstract

Promoting a transition to low-carbon energy systems to mitigate climate change requires an optimization of renewable energy (RE) planning. However, curated data for the most promising RE technologies, hydro-, wind and solar power, are missing, which limits data-based decision-making support. Here, a spatially explicit database for existing and proposed renewable power plants is provided: The Renewable Power Plant database for Africa (RePP Africa) encompasses 1074 hydro-, 1128 solar, and 276 wind power plant records. For each power plant, geographic coordinates, country, construction status, and capacity (in megawatt) are reported. The number of RePP Africa records exceeds the respective values in other existing open-access databases and matches available cumulative capacity data reported by international energy organizations best with deviations <13% for hydro-, <23% for wind, and <32% for solar power plants. This contemporary database is the most harmonized open-accessible reference source on RE power plants across Africa for stakeholders from science, (non-)governmental organizations, consulting, and industry; providing a fundamental data basis for the development of an integrated sustainable RE mix.

## Background & Summary

African countries face the challenge of providing electricity for 596 million people that currently lack access (i.e. 43% of actual population (1373 million); census: 2021)^1^. Concurrently, for advancing climate change mitigation and achieving long-term objectives of the Paris Agreement, the electricity sector needs to be climate-neutral by mid-century^2^. While many developed countries are dealing with the environmental impacts caused by a long history of fossil fuel energy dependence, African countries could leapfrog the transition to renewable, low-carbon energy supply systems^3,4^. In Africa, hydropower remains the primary renewable electricity resource, accounting for 70% of the renewable electricity share, which corresponds to 16% of total electricity production (2020)^5^. At the same time, the continent has the highest untapped hydropower potential worldwide, with actually 11% utilized^5^. An estimation of remaining potential for Africa amounts to 2.3 petawatt hours (PWh) per year, at costs of more than 0.5 US$ per kilowatt hour (kWh)^6^. Electricity generation from hydropower is considered renewable and one that can support climate change mitigation. However, costs and risks of large hydropower plants (HPPs) have often been underestimated while their benefits were overestimated^7^. HPPs operating with dams truncate natural river flows and may cause unexpected ecological, socioeconomic, and political ramifications on different temporal and spatial scales^8–11^. These changes are often irreversible. Furthermore, not only does dam building impact natural river systems, but dam removal can also cause unknown costs and ecological impacts on the formerly dammed river regimes^12,13^. Regarding the future performance of HPPs, climate change induced runoff changes (e.g., floods, prolonged droughts) are likely to jeopardize hydroelectricity generation in several African regions^14^. Other technologies to generate electricity from hydropower are run-of-river and pumped storage schemes. While run-of-river is a decade-old technology, with limited capacity for energy storage, pumped storage technology has gained increasing attention only very recently^15^. Consistent databases reporting and distinguishing the three technology types are still lacking. As a consequence, the opportunities for and impacts of these technology types on a national or continental level are poorly studied, constraining advanced hydropower energy planning^16^.

During recent years, solar and wind power have exhibited the highest growth rates among Africa’s renewable energy (RE) resources, yet they still contribute marginally to Africa’s energy resource mix (i.e. solar: 1.2%; wind: 1.5% share of total electricity generation in 2019^17^). Given the dependence of solar and wind power on meteorological variables, power generation from these RE resources is variable and intermittent, from short (sub-hourly) to long (seasonal and interannual) timescales^18^. In order to address this challenge, increased research attention has been given to approaches that investigate integrated RE storage options, to thereby exploit the complementary spatiotemporal properties of RE resources^19,20^.

Various databases on renewable power plants have been published. On a global scale, the Global Energy Observatory^21^, the Open Infrastructure Map^22^, and the Global Power Plant Database^23^ provide georeferenced information on fossil fuel and renewable power plants. Up to now, however, these open source databases lack information for Africa, in particular in the fast-developing domains of solar and wind power. The Global Dam and Reservoir Database (GranD) and the Future Hydropower and Reservoir Database (FHReD) are frequently cited as established databases reporting existing and future HPPs^24,25^; yet only HPPs operating with a dam and a reservoir are included. Published in 2021, the African Hydropower Atlas (AHA) presents a harmonized dataset on existing and planned hydropower plants to facilitate modelling of power systems across Africa^26^. At the same time, its restriction to hydropower plants limits renewable power plant modelling. In order to implement integrated modelling approaches on the (potential) electricity mix, and its implications in Africa, the African Energy Live Data database, provided by the African Energy company, has been increasingly used as a reliable source by the science community in the past^27–29^. However, the London based consultancy company only provides small shares of their data to the public and charges for further downloads for analysis processing^28^. The Wind Power is one of a few global databases that provides information on existing and proposed wind farms in Africa, but similar to African Energy Live Data, only parts of it are freely accessible^30^. For solar power plants the Wiki Solar database^31^ provides a similar service and covers globally more than 10,000 power plants. Again, data usage and replication are restricted and not available under a creative common license. The scientific use of renewable energy datasets without creative common license inflicts with the need that research published in scientific journals and including accessible datasets is reproducible. This lack of harmonized, open-access, and reliable datasets with georeferenced information on existing and proposed HPPs, solar power plants (SPPs), and wind power plants (WPPs) limits ongoing research efforts on the sustainable development of the energy resource mix and constrains a science-based discussion among stakeholders in the decision process.

In summary, two main approaches are currently used for renewable energy analyses on a continental or global scale: (1) Analyses are performed for one RE type, using a corresponding database^32^ or (2) integrated analyses for different RE types are performed and data is compiled from various databases^33,34^ or databases are behind paywalls^27^. The lack of a comprehensive and up-to-date database covering comparable information of existing and proposed HPPs, SPPs, and WPPs limits integrated renewable energy planning worldwide, in particular for Africa.

**Fig. 1:**
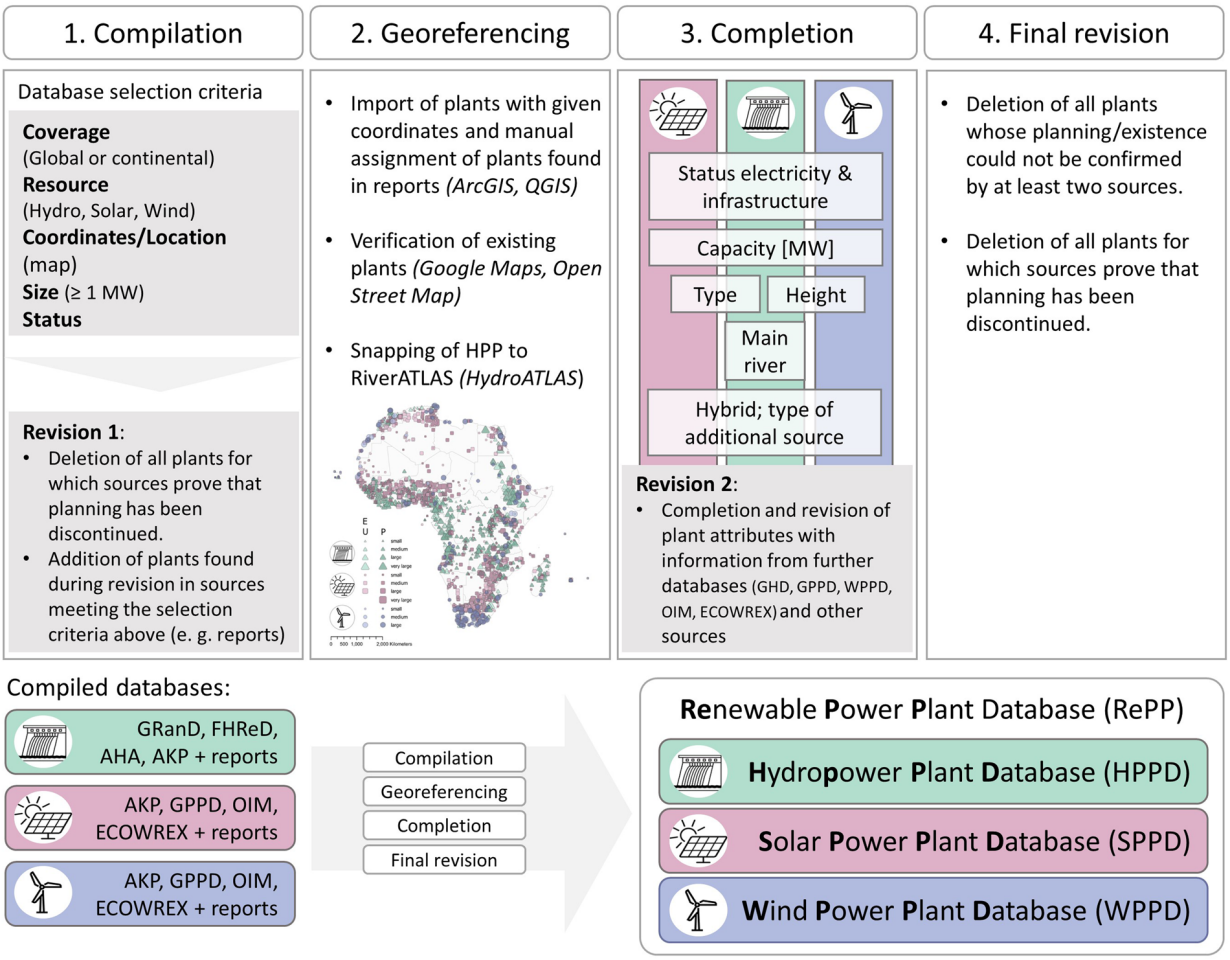
The four steps of the database generation process. 1. Compilation: Databases were selected based on five criteria: i. The database (DB) has a global or continental coverage; ii. The DB contains information on hydro, solar and/or wind power plants; iii. The DB provides coordinates or maps the location of power plants; iv. Power plants with a capacity of ≥1 megawatt (MW) are included; v. A status of the electricity production or plant facility is indicated (e. g. proposed/planned; under construction; existing). During revision 1 all compiled plants were revised to delete plants for which sources prove that planning has been discontinued and add plants that were identified in sources meeting the database selection criteria. 2. Georeferencing: All data records were georeferenced. 3. Completion: Summary of attributes that were checked for each plant to fill missing information. Background colours indicate if an attribute is given for all three RE types (pink = solar power, green = hydropower, blue = wind power) or only for one or two RE types. 4. Final revision: Data records with status cancelled or insufficient proof were deleted. The final product is the Renewable Power Plant Database (RePP) Africa which consists of the Hydropower Plant Database (HPPD), Solar Power Plant Database (SPPD), and Wind Power Plant Database (WPPD) for Africa.

Here, a comprehensive, curated and georeferenced renewable power plant database for Africa (RePP Africa) is presented (last revision: 16.11.2022)^35^. Data records were compiled and processed from various sources for all African countries. The establishment of the database included four steps: compilation, georeferencing, completion, and revision (Fig. 1).

The first openly-accessible and harmonized renewable power plant database covering entire Africa includes georeferenced information on a total of 1074 HPPs, 1128 SPPs, and 276 WPPs. 401 HPPs, 411 SPPs, and 127 WPPs are existing or under construction, with a total capacity of 59.56 gigawatts (GW), 10.56 GW, and 10.53 GW respectively (Table 1). As of November 2022, 673 HPPs, 717 SPPs, and 149 WPPs are proposed with a total respective capacity of 130.85 GW, 53.32 GW, and 16.87 GW.

**Table 1:** Summary of number and capacity (MW) of hydro-, solar, and wind power plants according to four size categories.

Summaries are given for the status of the power plant facility (stat_inf) distinguishing existing plants (E), plants under construction (U) and proposed plants (P). Numbers in brackets: Percentage [%] of the total.

RePP lists three types of power plant facility status (status_inf): existing (E), under construction (U), and proposed (P). Proposed plants include potential sites where feasibility studies were realized. Once the construction has started, the status changes to under construction. If a plant is officially inaugurated, its status turns to existing. Each data entry is provided with a time stamp indicating when the status was last checked.

Power plant facilities might exist but not generate electricity for uncertain time periods due to destruction or other reasons. However, the searched databases do not distinguish operating and temporarily not operating existing plants. In order to enable RePP Africa users to consider this issue when using RePP Africa data^35^, we include the status of electricity generation (status_ele). It gives information on the status of power generation and distinguishes between operating (O), under construction (U), proposed (P), and not operating (NO). Since in the last case, the infrastructure of the power plant facility is existing, not operating plants could be rehabilitated and operate again.

For HPPs and SPPs, different operating systems are distinguished. 446 HPPs operate with reservoir storage, 286 as run-of-river HPPs, and 17 HPPs with a pumped-storage system. No adequate information could be provided for 325 HPPs (30% of total), with 86% of these categorized as proposed (281 HPPs). Most SPPs (1072) are operating or proposed as photovoltaic (PV), 47 as concentrated solar power (CSP), and 9 as concentrator photovoltaics (CPV) type plants.

**Fig. 2:**
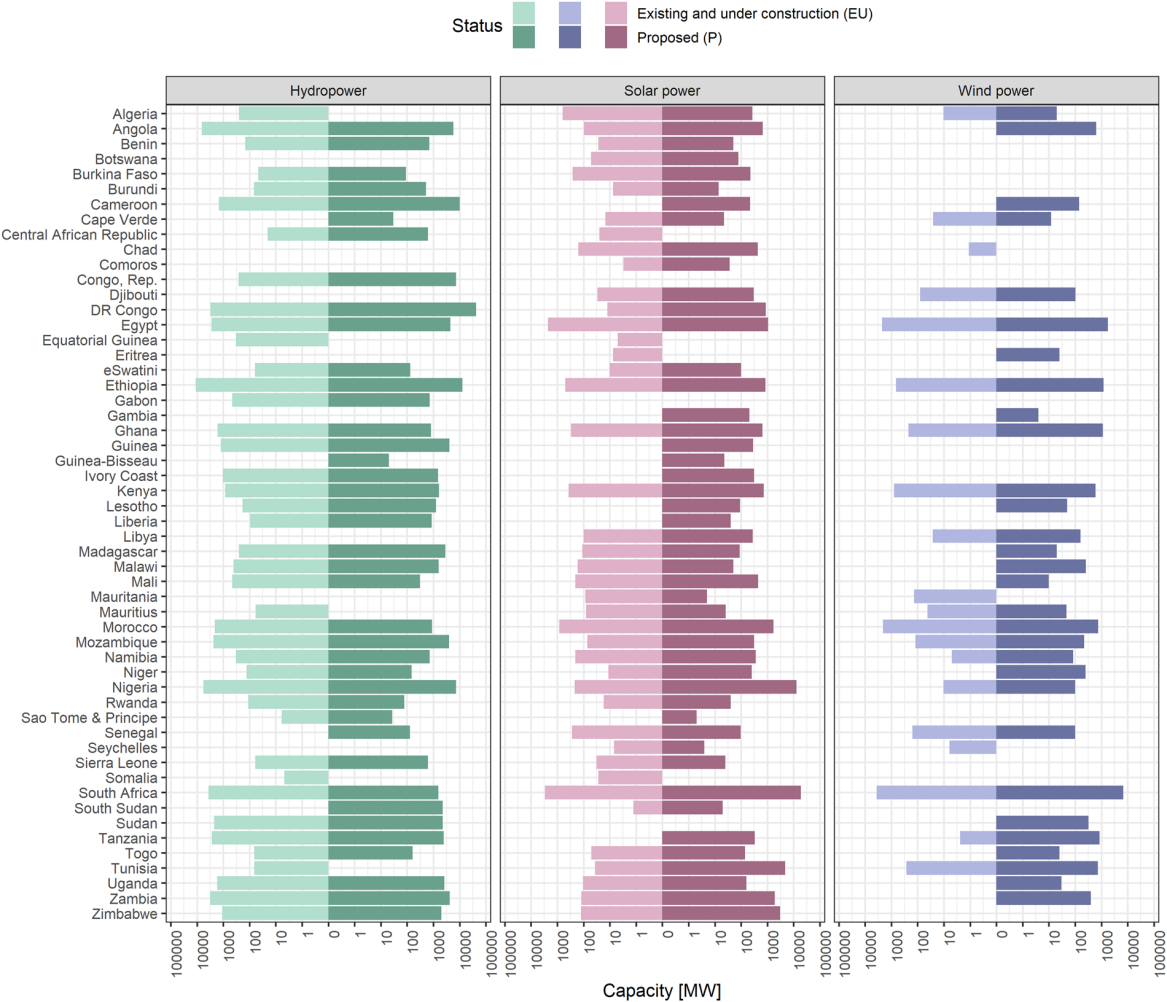
Cumulated capacity in megawatts [MW] by country for hydropower, wind power, and solar power plants. In this figure, the Sahrawi Arab Democratic Republic (Western Sahara) is geographically aggregated with Morocco. No statements on the political situation are intended.

As of November 2022, all 55 African countries have installed or proposed energy generation capacity from RE resources. Solar power and wind power are playing an increasingly important role in the total RE resource mix, with shifts between installed and proposed total capacity differing among countries (Fig. 2).

The contemporary, curated database on renewable power plants (existing, under construction, proposed) in African countries will enable the research community to address and fill current research gaps and to advance integrated renewable energy modelling. Openly accessible data on renewable energy plants vary in quality among countries. The RePP Africa^35^ intends to stimulate integrated research and large-scale assessments at a continental level as well as to foster case studies and research activities in data-poor regions of less-studied African countries.

## Methods

The spatially-explicit, renewable powerplant database for Africa (RePP Africa^35^) aims to advance existing efforts in the field of open-accessible renewable energy data. By harmonizing, reviewing, and updating information on power plants from established databases^21,23^ and adding information from other sources, RePP Africa^35^ is a coherent database on the current key technologies hydro-, solar and wind power for the African continent. RePP was created to respond to an increasing demand for datasets that allow integrated electricity planning and impact assessment modelling on renewable power technologies. RePP Africa^35^ is composed of three sub-datasets, containing information for hydropower, solar power and wind power. This approach enables the user to apply the database either for a specific energy source or across RE sources. RePP Africa^35^ covers all African countries and includes existing power plants (E), plants under construction (U) and proposed power plants (P). It further indicates the status of electricity production, because existing power plant facilities might be temporarily out of operation (operating (O), under construction (U), proposed (P), not operating (NO). Data collection started in March 2021. The last revision was finished in November 2022. Power plants scheduled to go into operation in 2022 are kept in the categories “under construction” or “proposed” if no source indicating inauguration or construction start is given. However, continuous revisions will be performed in order to update the database and to adjust information if necessary or justified.

### Compilation

Data was collected from a wide array of available information sources. First, data records on existing and proposed projects were compiled from available databases with creative common license (Fig. 1). Databases had to match the following criteria: (1) Coverage of the database is global or continental (Africa). Due to limited availability of freely accessible databases for wind and solar power plants we optionally accepted the ECOWAS Observatory for Renewable Energy and Energy Efficiency (ECOWREX)^36^ covering West Africa. (2) The database includes information on hydropower, solar power, or wind power plants: The database contains information on all RE resources or, in selected cases, only on a specific energy source. (3) The database includes precise geographic information on the power plant location, ideally coordinates (latitude, longitude), or is displayed in form of a freely-accessible map that indicates plant location. In the latter case, coordinates were manually derived using ArcGIS Pro^37^ and QGIS^38^. (4) The database provides the capacity of the respective plant in megawatts [MW], at least for the majority of the power plant projects. Reservoir size data in million cubic meters [mcm] from the Global Reservoir and Dam Database (GRanD)^24^ was optionally accepted. (5) Information on the status of electricity production (not operating, operating, under construction, proposed) or the power plant facility (existing, under construction, proposed) is provided. The Africa Knowledge Platform (AKP)^39^ provides capacity values in megawatts only for existing plants, which was optionally accepted because AKP was the database with the highest number of data records for all renewable resources. Data entries were combined with further information found during the following revision steps.

Four databases were used to compile information of power plants for each renewable source: For hydropower plants we used information from the Global Reservoir and Dams Database v1.3 (GRanD)^24^, the Future Hydropower and Reservoir Database (FHReD)^25^, the African Hydropower Atlas v2.0 (AHA)^26^, and Power Plants by the Africa Knowledge Platform^39^. For solar and wind power we used information from Power Plants by the Africa Knowledge Platform^39^, the Global Power Plant Database v1.3.0 (GPPD)^23^, ECOWAS Observatory for Renewable Energy and Energy Efficiency (ECOWREX)^36^, and Open Infrastructure Map^22^. After collection and harmonizing power plant information for each resource in an Excel sheet, all data entries were revised using Google search engine (Revision 1). Entries of power plants for which sources prove that planning has been discontinued were deleted. If the database compilation revealed inconsistent information, we consulted further sources in order to assure providing a correct information for each plant. Therefore, we prioritized (1) information from individual, specific project reports and (2) information from up-to-date sources (referring to the current timestamp of the data entry). Inconsistent information was mainly found for proposed power plants, because feasibility studies and project proposals do not assure one capacity value to be installed but present different capacity values. For the attribute g_cap_mw (given capacity in megawatts (MW)), we selected the capacity indicated by the majority of sources and saved further information in the field “other capacity” (other_cap_mw). Additional power plants found during revision 1 in other literature sources matching criteria (3), (4), and (5) were added. Only openly-accessible information from databases with creative common license was included. All licences that apply to the searched databases are listed in RePP Africa^35^.

### Georeferencing

All plants with given coordinates were imported to ArcGIS Pro^37^ and QGIS^38^ software. All plants with locations indicated or described by reports or other sources were added manually and coordinates checked for plausibility. Existing plant locations were cross-checked using Google Maps and Open Street Map and, if necessary, corrected. All HPPs were snapped to river lines of the RiverATLAS (HydroATLAS)^35^ to facilitate further processing.

### Completion

In order to complete information, all plants were revised (revision 2) using Google search engine and information from further databases (Hydropower: Global Hydropower Database^40^, Global Power Plant Database (GPPD)^23^, World Power Plant Database (WPPD)^21^, Open Infrastructure Map^22^, and ECOWAS Observatory for Renewable Energy and Energy Efficiency (ECOWREX)^36^; Solar and Wind Power: World Power Plant Database (WPPD)^21^. The database Power Africa from the African Development was excluded during last revision in November 2022, because the database is no longer accessible due to unknown reasons. Table 2 summarizes all available attributes and the relative proportion [%] of plants for which the information is available. In addition, RePP Africa^35^ indicates for each plant if it is listed in one of the consulted databases and specifies further used sources. Such additional data sources can be divided into three categories: (1) summary reports and books that list power plants on a regional/national scale including all three power types or on a continental/global level for a specific power source; (2) information pertaining to individual projects, from technical project fact sheets, environmental impact assessments, peer-reviewed papers, and project summaries or presentations from engineering companies; and (3) online newspaper articles and social media announcements.

**Table 2:** Overview of attributes (metadata) provided in the African renewable power plant database (RePP Africa).

Attribute (column name) and a description are given. For each renewable type (hydropower plant database (HPPD), solar power plant database (SPPD), and wind power plant database (WPPD)) the share of records that have a data entry for this attribute is indicated as percentage [%] of the total number of records (i.e., 100% indicates that the attribute is given for all plants). If attributes are not available for all data records (<100%), the relative proportion of available records is given in percent [%] for all plants and for existing and under construction/proposed plants separately (in brackets).

### Final revision

Each plant record was revised by the authors and specifically checked on plant existence or proposal. To increase data quality and exclude outdated and incorrect data records, each entry is confirmed by two or more references. In order to assure the latter, we consulted sources found via Google search engine and freely accessible data from African Energy Live Data^28^, The Wind Power^30^, and Wiki-Solar^31^. The consultation of these databases was in aggreement with their respective terms of use: No information was downloaded or copied and only freely accessible infromation was consulted to confirm information collated for RePP Africa^35^. Collected data records were excluded when (1) a second source was not found during final revision or (2) when the proposed plant was irreversibly cancelled. Date of last revision and edit is indicated for each plant and for the complete database. Last revision of the complete current version was November 2022. Just before RePP Africa^35^ database submission, 5% of all database entries were randomly selected (49 HPP, 67 SPP, and 8 WPP power plants) and carefully checked again against the defined selection criteria as mentioned above to provide a most updated database version and get an idea about the potential error in the data (e.g. due to typos or invalid links to online references). This final check was conducted between November 16 and 17, 2022, and did not show any errors in the selected entries.

## Data Records

**Fig. 3:**
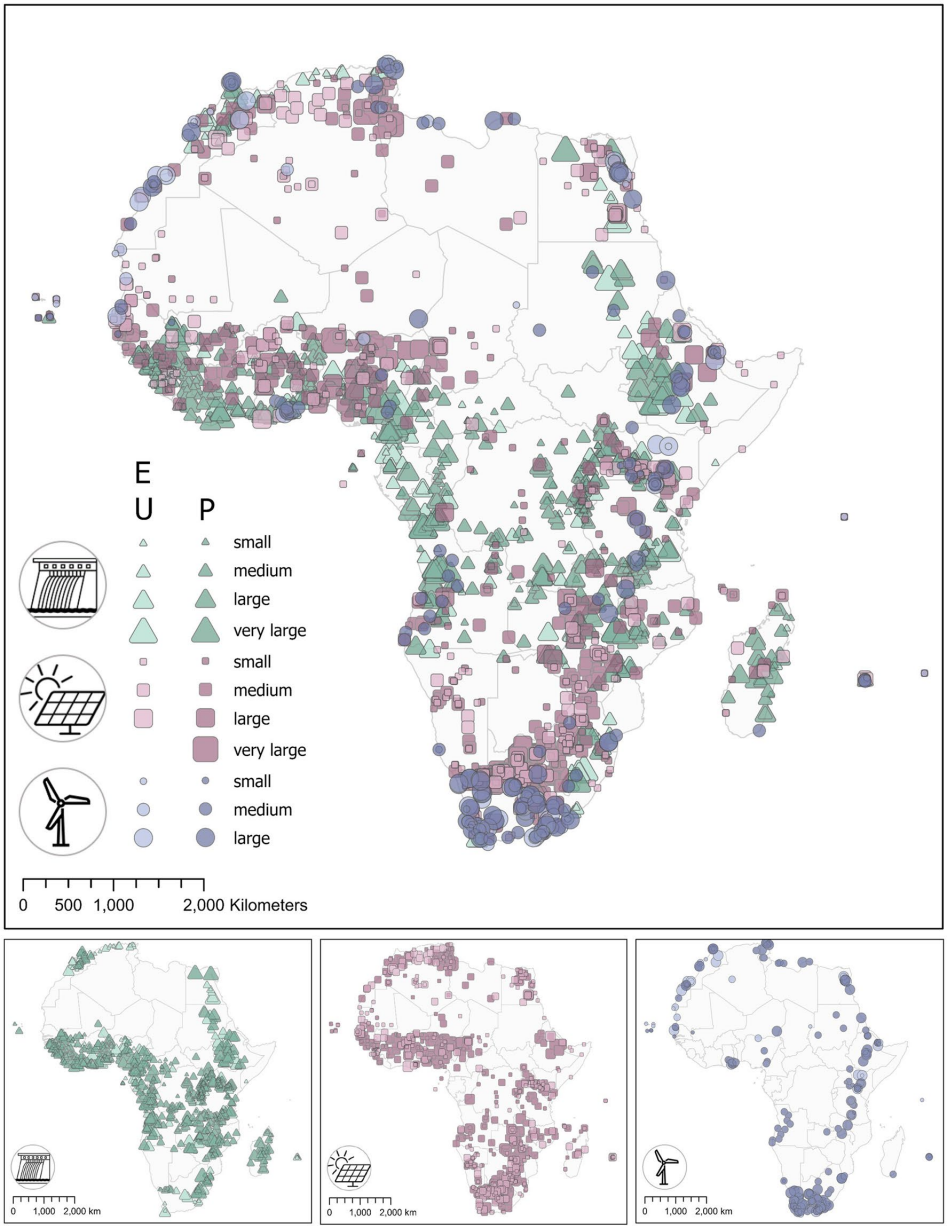
Map of all hydropower, solar power and wind power plants as compiled in the African Renewable Power Plant database (RePP Africa). Symbol colour and shape indicate renewable energy type; colour intensity indicates status (E - existing and U - under construction, P - proposed); symbol size indicates capacity in megawatts [MW] with small 1–10 MW, medium >10–100 MW, large >100–1000 MW, and very large >1000 MW. No plants are located on open water; all facilities not located on the mainland are located on islands.

RePP Africa^35^ is collated in a single spreadsheet-based file and consists of the hydropower plant database (HPPD), solar power plant database (SPPD), and wind power plant database (WPPD). Figure 3 maps all data records according to resource type (symbol colour, symbol shape) and capacity, i.e. size in megawatts (symbol size).

**Fig. 4:**
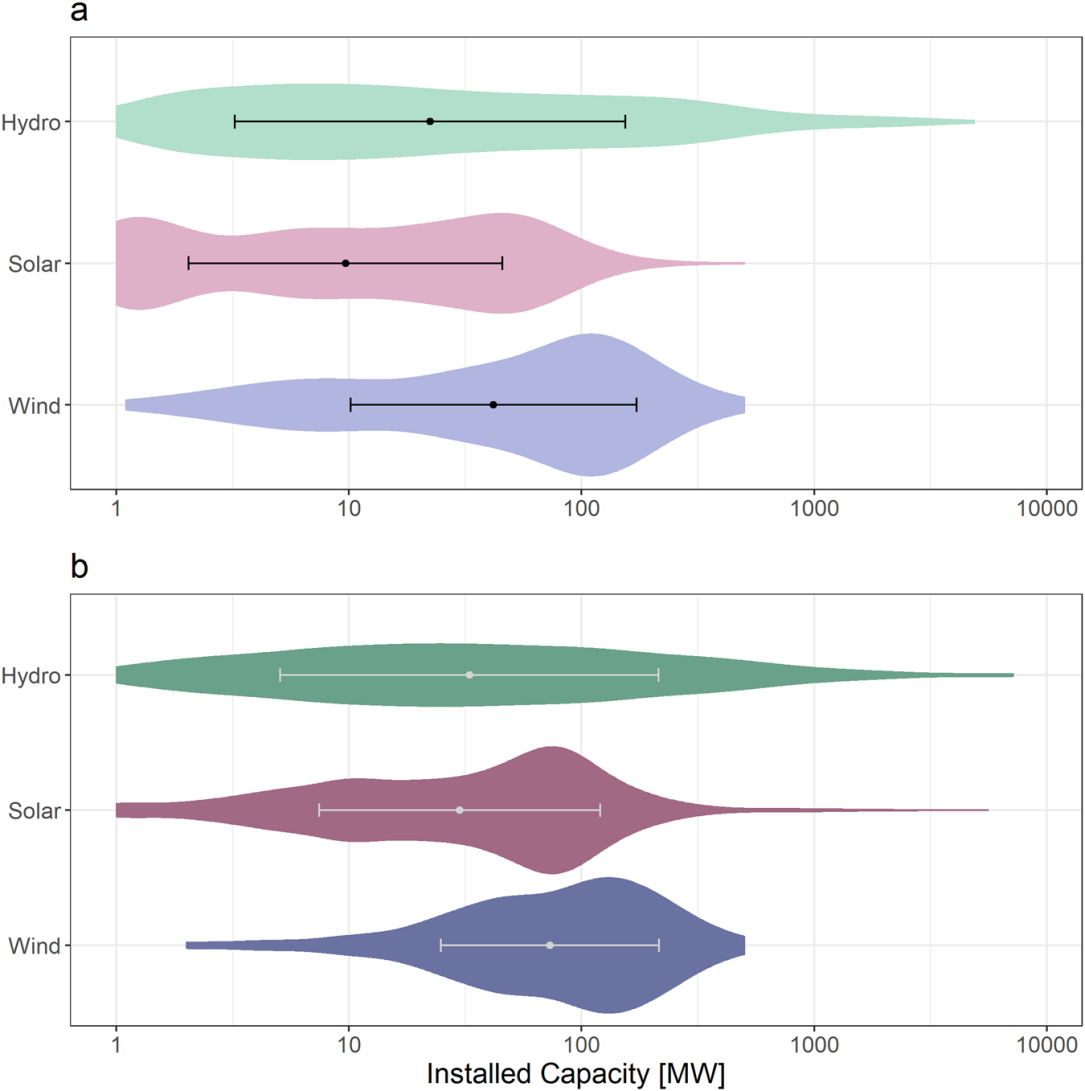
Distribution of RePP data entries by installed capacity in megawatts (MW) and status (stat_inf) for hydropower (green), solar power (pink) and wind power (blue). (**a**) Existing plants and plants under construction. (**b**) Proposed plants.

RePP Africa^35^ provides data records for hydropower, solar power, and wind power plants in all African countries (Fig. 3). The database is hosted on figshare^35^. The repository includes one Excel file with eleven sheets containing one information sheet on the general structure of the file and the sheets included (Info), one overarching sheet with metadata (S1) and, for each of the three RE resources (hydro, solar-, wind power), three specific sheets that provide (1) the RE specific metadata (S2, S5, S8), (2) the respective dataset (S3, S6, S9), and (3) the data sources (S4, S7, S10). The overarching table with metadata gives an overview on all attributes including descriptions of attributes and the number of plants for which an attribute is reported (Table 2). Figure 4 summarizes the distribution of RePP Africa data entries by installed capacity for existing plants and plants under construction, and proposed plants.

**Fig. 5:**
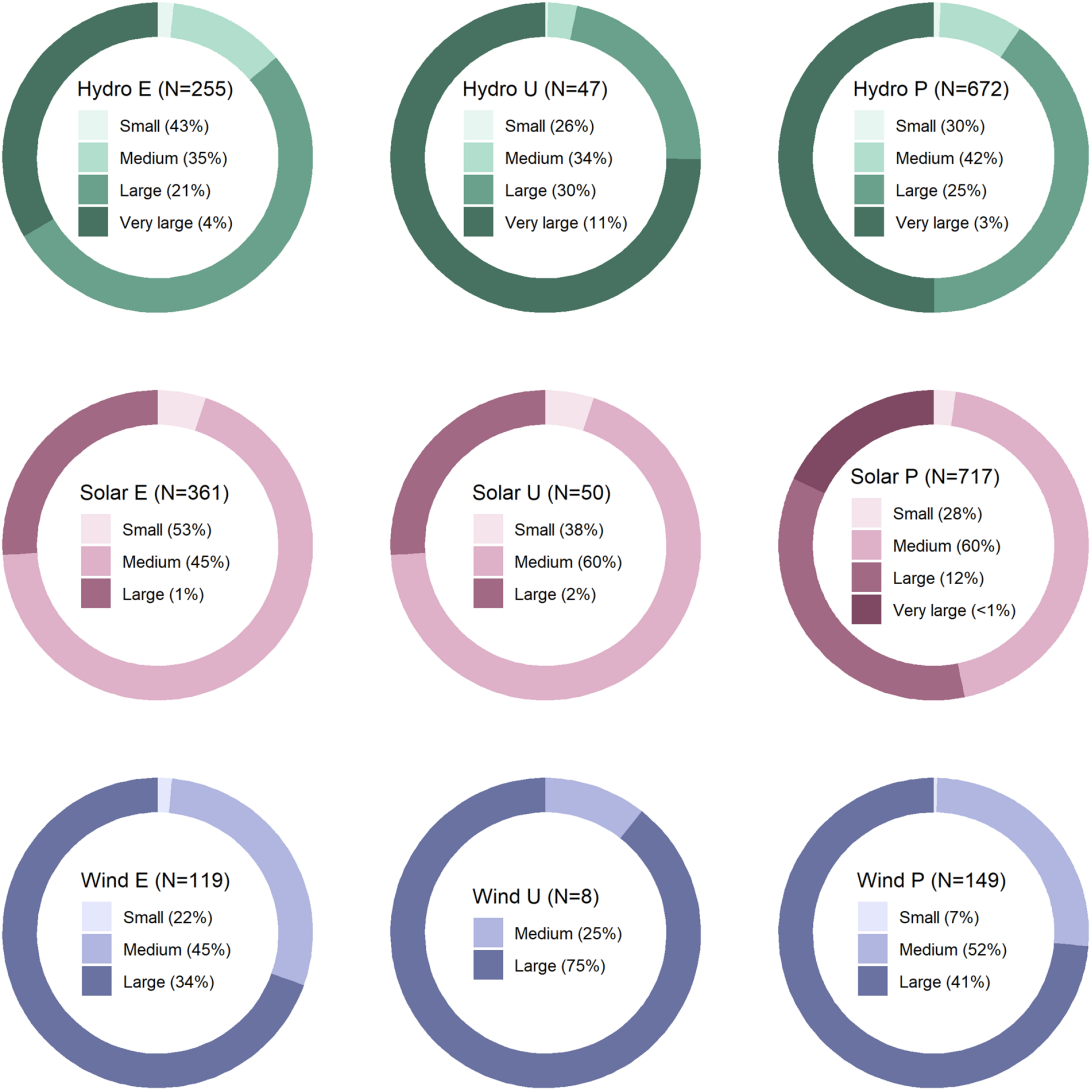
Share of total capacity in megawatts (donut plots) for existing plants (E), plants under construction (U), and proposed plants (P) for hydropower (green), solar power (pink), and wind power (blue). Number of plants [N] and shares in percent [%] of N are annotated.

RePP Africa^35^ data entries were further aggregated for each resource type in four capacity size categories to illustrate differences between share of capacity and number of existing and proposed power plants (Fig. 5). We differentiated between small (1–10 MW), medium (>10–100 MW), large (>100–1000 MW), and very large (>1000 MW) power plants.

During the first step of data compilation (Fig. 1), several plants of the searched databases were rejected and not compiled due to varying reasons: (1) The capacity was below 1 MW. (2) No location was indicated. (3) Power plant only occurred in one of the databases that were searched and no further information was found (including sources used for revision only). (4) No capacity was indicated. (5) No name was indicated (occurred only for plants compiled from Open Infrastructure Map^22^). Table 3 gives on overview on the number of plants that were disregarded after the first search of the databases.

**Table 3:** Total number of power plants of the searched databases for each resource type.

Number of rejected power plants are indicated per country and database for each resource type (hydropower, solar power, wind power). The total number of rejected power plants is indicated. ‘ -’ indicates that the database has no data entry for the respective country. Grey coloured cells indicate that RePP Africa does not list any power plants for the respective resource. The approximate number of included power plants was calculated as the difference between total number of power plants and total number of rejected power plants per database and renewable resource. The absolute number of plants in RePP Africa can differ from the calculated value due to differences (e. g. duplications or different counting of plant phases) between RePP Africa and searched databases. Database abbreviations: AKP – Africa knowledge platform^39^; AHA – African Hydropower Atlas^26^; FHReD – Future Hydropower Reservoir and Dams Database^25^; GRanD – Global Reservoir and Dams database (main purpose: hydroelectricity)^24^; GPPD –Global Power Plant Database^23^; ECOWREX - ECOWAS Observatory for Renewable Energy and Energy Efficiency^36^; OIM - Open Infrastructure Map^22^. In this figure, the Sahrawi Arab Democratic Republic (Western Sahara) is geographically aggregated with Morocco. No statements on the political situation are intended.

## Technical Validation

RePP Africa^35^ compiles and revises existing data on hydropower, solar power and wind power for the entire African continent. The presented openly accessible and curated database is an attempt to meet the demand of the renewable energy modelling science community for free, accurate, and harmonized scientific data. All data and related information have been checked several times and are confirmed by at least two references each. Limitations in the accuracy of locations and other attributes might occur in particular for proposed power plants. We performed a technical validation for RePP Africa^35^ by randomly selecting 5% of all data entries after the last full revision, which ended on 16 November 2022. The random selection resulted in a dataset of 128 data entries (49 hydro-, 67 solar, and 8 wind power plants). We checked all attributes and sources. All data entries where consistent with the all or the majority of provided sources. We did not find inconsistent or missing data entries or non-functioning links. Each reported status (stat_inf) was in line with given sources and all indicated databases were correct. For 6%, minor discrepancies were found (duplicate source (1), one of all sources refers to a different plant (5), majority of sources indicates a slightly different capacity (2). However, despite these discrepancies, the majority of sources still confirmed the given information and in case of differing capacity values the difference was acceptable (<5%).

Outstanding in comparison to other databases is the number of references cited for each data record: 36% of all hydro-, 77% of all solar, and 59% of all wind power plant records are validated by two or three sources, the rest is validated by up to 15 references (Table 4).

**Table 4:** Total number of data entries for all sources (summary databases & additional sources) and for the different data generation process steps (Fig. 1): Compilation databases for 1.

Compilation; Completion databases for 3. Completion, Revision 2; Further sources for 1. Compilation, Revision 1; Revision databases for 4. Final revision. Percentages refer to the total number of power plants per resource (Hydropower N = 1074, Solar power N = 1128, Wind power N = 276).

In total, 1172 different references are provided for HPPs, 1134 for SPPs, and 432 for WPPs. 33% was collected from website and newspaper articles, 20% from development or environmental reports, company or government power point presentations, and fact-sheets, 16% from other databases, 14% from company websites, and 10% from Encyclopaedia records. Less frequently, information from peer-reviewed articles (3%), UN, development bank, and government websites (2%), social media (1%), and books or theses (1%) was obtained.

Compiled total numbers and capacities of the different renewable power plant types have been cross-checked with available cumulated continent-related data (Table 3).

In comparison to other currently existing open-access databases, RePP Africa^35^ lists more renewable power plants with cumulated capacity better matching the respective values reported by IHA^41^, IEA^42^, and IRENA^43^ (Table 5). It highlights that we could enhance quality and completeness of RePP Africa^35^ by not only searching existing databases but also including plants from additional sources (compilation, revision 1). The latter applies in particular to wind and solar power plants. In general, the cumulated capacities of existing power plants included in the RePP Africa^35^ differ by minimum + 3.07% (hydropower) and maximum −31.82% (solar power) from data reported by IHA, IEA, IRENA, and Hydropower & DAMS^44^ for 2020 and 2021 (Table 5, HPPD: +3.07% (IHA 2021), +3.98% (IRENA 2021), +12.81% (H&D 2020); SPPD: −27.31% (IEA 2021), −31.82% (IRENA 2021); WPPD: +12.86% (IEA 2021) +23.19 (IRENA 2021)). The largest discrepancy is between reported cumulated capacity of proposed HPPs with 132.05 GW according to the here presented database (HPPD) and 49–115 GW in the available literature (Hydropower & DAMS). The following reasons may be explanations for the discrepancies in the hydropower data: (1) The HPPD contains data records on HPPs not covered by IHA or Hydropower & DAMS; (2) The HPPD includes up-to-date data (2021) which is so far not included in IHA or Hydropower & DAMS datasets from 2020; (3) The implementation of the proposed HPP is subject to uncertainty. Different organisations use different definitions when including proposed or planned power plants in cumulative capacity calculations. Underestimation of the cumulated solar power capacity in RePP Africa^35^ in comparison to IEA and IRENA might result from neglecting all solar power plants <1 MW. Another discrepancy comes up when taking a look at the details of the different solar power plant types. IEA further differentiates between solar power from photovoltaics (PV) and concentrated solar power (CSP). According to them, in 2021, 9.4 GW is installed as PV and 1.4 as CSP versus 7 GW as PV and 3 GW as CSP, respectively, in the SPPD of RePP Africa^35^.

**Table 5:** Comparison of data from all searched databases (compilation databases) to the here presented African Renewable Power Plant Database (RePP Africa) and to other established data sources for renewable energy assessment (validation databases, year of census in brackets).

Number of plants and cumulated capacities for the whole African continent are given in gigawatts (GW) for the specific renewable resources (hydropower, solar power, wind power) and the specific construction status (existing (E), under construction (U), proposed (P)). AKP – Africa Knowledge Platform; AHA v2.0 – African Hydropower Atlas Version 2.0; GRanD v1.3 – Global Reservoir and Dams Database Version 1.3; FHReD – Future Hydropower Reservoirs and Dams Database; GPPD v1.3.0 – Global Power Plant Database Version 1.3.0; ECOWREX - ECOWAS observatory for Renewable Energy and Energy Efficiency; OIM – Open Infrastructure Map; IHA – The International Hydropower Association; IEA – The International Energy Agency; IRENA – The International Renewable Energy Agency; GWED – The Global Wind Energy Council. Empty fields: No data.

At present, wind and solar energy outpace hydropower and other renewable sectors with their growth rates. Although the IEA provides forecasts for cumulative capacities for 2021 and 2022, these analyses are based on data from 2020, making the presented database RePP Africa^35^ the most up-to-date open-access database on renewable power plants in Africa.

## Data Availability

All processing steps including data compilation and georeferencing were realized with ArcGIS Pro 2.9.5 software from ESRI^37^. The open-source software QGIS version 3.18 was used by assistants as additional software to georeferenced power plant locations^38^. We used the ArcGIS ‘*Edit – Create’* function and the QGIS ‘A*dd feature’* function to manually assign power plant locations in cases where maps but no coordinates were accessible. Additional information is described in detail in the Material and Methods section. No stand-alone programming code was created.

## References

[1] IEA. *The pandemic continues to slow progress towards universal energy access*. https://www.iea.org/commentaries/the-pandemic-continues-to-slow-progress-towards-universal-energy-access (2021).

[2] Kuramochi T (2018). Ten key short-term sectoral benchmarks to limit warming to 1.5 C. Clim. Policy.

[3] Mutezo G, Mulopo J (2021). A review of Africa’s transition from fossil fuels to renewable energy using circular economy principles. Renew. Sustain. Energy Rev..

[4] Momodu AS, Okunade ID, Adepoju TD (2022). Decarbonising the electric power sectors in sub-Saharan Africa as a climate action: A systematic review. Environ. Challenges.

[5] International Hydropower Association (IHA). *2021 Hydropower Status Report, Sector trends and insights*. https://www.hydropower.org/status-report (2021).

[6] Gernaat DEHJ, Bogaart PW, Vuuren DPV, Biemans H, Niessink R (2017). High-resolution assessment of global technical and economic hydropower potential. Nat. Energy.

[7] Moran EF, Lopez MC, Moore N, Müller N, Hyndman DW (2018). Sustainable hydropower in the 21st century. Proc. Natl. Acad. Sci..

[8] Peters R (2021). Integrated impact assessment for sustainable hydropower planning in the Vjosa Catchment (Greece, Albania). Sustainability.

[9] Zarfl C (2019). Future large hydropower dams impact global freshwater megafauna. Sci. Rep..

[10] De Faria FAM, Davis A, Severnini E, Jaramillo P (2017). The local socio-economic impacts of large hydropower plant development in a developing country. Energy Econ..

[11] Thieme, M. L. *et al*. Navigating trade-offs between dams and river conservation. *Glob. Sustain*. **4** (2021).

[12] Bellmore JR (2019). Conceptualizing Ecological Responses to Dam Removal: If You Remove It, What’s to Come?. Bioscience.

[13] Habel M (2020). Dam and reservoir removal projects: a mix of social-ecological trends and cost-cutting attitudes. Sci. Rep..

[14] Van Vliet MTH (2016). Multi-model assessment of global hydropower and cooling water discharge potential under climate change. Glob. Environ. Chang..

[15] Hunt JD (2020). Global resource potential of seasonal pumped hydropower storage for energy and water storage. Nat. Commun..

[16] Kuriqi A, Pinheiro AN, Sordo-Ward A, Bejarano MD (2021). & Garrote, L. Ecological impacts of run-of-river hydropower plants—Current status and future prospects on the brink of energy transition. Renew. Sustain. Energy Rev..

[17] IRENA. *Renewable Energy Market Analysis: Africa and Its Regions*. https://www.irena.org/publications/2022/Jan/Renewable-Energy-Market-Analysis-Africa (2022).

[18] Engeland K (2017). Space-time variability of climate variables and intermittent renewable electricity production – A review. Renew. Sustain. Energy Rev..

[19] Sterl S (2021). A Grid for all Seasons: Enhancing the Integration of Variable Solar and Wind Power in Electricity Systems Across Africa. Curr. Sustain. Energy Reports.

[20] Solomon AA, Child M, Caldera U, Breyer C (2020). Exploiting wind-solar resource complementarity to reduce energy storage need. AIMS Energy.

[21] Global Energy Observatory (GEO). *World Power Plant Database*. http://globalenergyobservatory.org/ (2022).

[22] OpenStreetMap. *Open Infrastructure Map*. https://openinframap.org/ (2022).

[23] Byers, L. *et al*. *A global database of power plants*. https://www.wri.org/publication/global-database-power-plants (2018).

[24] Lehner B (2011). High-resolution mapping of the world’s reservoirs and dams for sustainable river-flow management. Front. Ecol. Environ..

[25] Zarfl C, Lumsdon AE, Berlekamp J, Tydecks L, Tockner K (2015). A global boom in hydropower dam construction. Aquat. Sci..

[26] Sterl S (2021). A spatiotemporal atlas of hydropower in Africa for energy modelling purposes. Open Res. Eur..

[27] Alova G, Trotter PA, Money A (2021). A machine-learning approach to predicting Africa’s electricity mix based on planned power plants and their chances of success. Nat. Energy.

[28] African Energy. *African Energy Live Data*. https://www.africa-energy.com/live-data (2022).

[29] Mahmood H, Alkhateeb TTY, Furqan M (2020). Exports, imports, foreign direct investment and CO2 emissions in North Africa: Spatial analysis. Energy Rep..

[30] Pierrot, M. *The Wind Power*. https://www.thewindpower.net/ (2022).

[31] WolfeWare. *Wiki-Solar*. https://www.wiki-solar.org/map/continent/index.html?Africa?0?f (2022).

[32] Thieme ML (2020). Dams and protected areas: Quantifying the spatial and temporal extent of global dam construction within protected areas. Conserv. Lett..

[33] De Angelis P (2021). Data-driven appraisal of renewable energy potentials for sustainable freshwater production in Africa. Renew. Sustain. Energy Rev..

[34] Sterl S (2020). Smart renewable electricity portfolios in West Africa. Nat. Sustain..

[35] Peters R, Berlekamp J, Tockner K, Zarfl C (2022). Figshare.

[36] ECREE. *ECOWREX.* http://www.ecowrex.org/mapView/index.php?lang=eng (2022).

[37] ESRI Inc. ArcGIS Pro version 2.9.5. *Esri Inc.* https://www.esri.com/en-us/arcgis/products/arcgis-pro/overview (2022).

[38] QGIS Development Team. QGIS Geographic Information System, version 3.18. Open Source Geospatial Foundation Project https://qgis.org/en/site/ (2021).

[39] European Commission. *Africa Knowledge Platform - Power plants*. https://africa-knowledge-platform.ec.europa.eu/ (2020).

[40] Wan, W., Zhao, J., Popat, E., Herbert, C. & Döll, P. Analyzing the Impact of Streamflow Drought on Hydroelectricity Production: A Global-Scale Study. *Water Resour. Res*. **57**, (2021).

[41] International Hydropower Association (IHA). *2022 Hydropower Status Report - Sector trends and insights*. https://www.hydropower.org/publications/2022-hydropower-status-report (2022).

[42] IEA. *Renewables 2021*. https://www.iea.org/reports/renewables-2021 (2021).

[43] IRENA. *Renewable energy statistics 2022*. https://www.irena.org/publications/2022/Jul/Renewable-Energy-Statistics-2022 (2022).

[44] Hydropower and DAMS. *World Atlas & Industry Guide 2021*. https://www.hydropower-dams.com/world-atlas/ (2021).

